# Experimental Investigations on Bond Behavior between FRP Bars and Advanced Sustainable Concrete

**DOI:** 10.3390/polym14061132

**Published:** 2022-03-11

**Authors:** Yingwu Zhou, Guojian Wu, Limiao Li, Zhipei Guan, Menghuan Guo, Lei Yang, Zongjun Li

**Affiliations:** 1 Guangdong Provincial Key Laboratory of Durability for Marine Civil Engineering, Shenzhen University, Shenzhen 518060, China; ywzhou@szu.edu.cn (Y.Z.); wuguojian2019@email.szu.edu.cn (G.W.); 1900471002@email.szu.edu.cn (L.L.); 1810332001@email.szu.edu.cn (Z.G.); menghuan.guo@szu.edu.cn (M.G.); yanglei@szu.edu.cn (L.Y.); 2 Key Laboratory for Resilient Infrastructures of Coastal Cities (MOE), Ministry of Education, Shenzhen 518060, China

**Keywords:** advanced sustainable concrete (ASC), fiber-reinforced polymer (CFRP) bars, limestone calcined clay cement (LC3), bond behavior, constitutive model

## Abstract

In response to resource shortage and carbon dioxide emissions, an innovative type of sustainable concrete containing LC3, seawater, sea sand, and surface-treated recycled aggregates is proposed in this study to replace traditional concrete. To understand the bond properties between the sustainable concrete and CFRP bars, an investigation was conducted on the bond behavior between sand-coated CFRP bars and advanced sustainable concrete. Pull-out tests were carried out to reveal the failure mechanisms and performance of this bond behavior. The results showed that the slip increased monotonically along with the increase in confinement. The bond strength increased up to approximately 15 MPa, and the critical ratio of C/D was reached. The critical ratio approached 3.5 for the Portland cement groups, while the ratio was determined as approximately 4.5 when LC3 was introduced. When the proportion of LC3 reached 50%, there was a reduction in bond strength. A multisegmented modified bond–slip model was developed to describe the four-stage bond behavior. In terms of bond strength and slip, the proposed advanced concrete exhibited almost identical bond behavior to other types of concrete.

## 1. Introduction

In many ways, durable, tailorable, and cost-effective concrete structures are desirable for the construction industry. Worldwide, approximately 30 billion tons of concrete is consumed annually [1]. The demand for such material has outpaced other construction substances and shown an ever-growing trend. Such an extensive consumption of concrete has led to two major environmental consequences: heavy exploitation of natural resources and excessive carbon dioxide emissions.

Typically, traditional concrete, also known as ordinary Portland cement (OPC) concrete, is produced by adding fresh water to cement. When in contact, it creates “gels” that hold river sand and gravel together into concrete [2]. The massive exploitation of the components from nature, such as fresh water, fine aggregates (river sand), and coarse aggregates (rock fragments), is likely to result in a crisis of natural resources, particularly in coastal regions, where the shortage of natural resources has become an inevitable concern for human beings. It is therefore important to search for reliable alternatives for each of the individual components. Continued efforts have been made accordingly to address the shortage of natural resources, such as geopolymer concrete [3,4] and recycled aggregates [5,6]. Recently, an innovative concept of seawater sea sand recycled aggregate concrete has been proposed by many researchers [7,8,9,10,11], in which seawater, sea sand, and recycled aggregates are applied collectively as replacements for fresh water, river sand, and rock fragments. These more sustainable alternatives have been certified as successful substitutes.

Excessive carbon dioxide emissions are inevitable from the manufacture of Portland cement during lime calcination and the concomitant fuel combustion. It has been reported that the manufacture of cement is responsible for 8% of global emissions [12]. Hence, an alternative cement with the long-term effect of low carbonization is urgent. Indeed, industrial disposals, such as fly ash and slag, are considered partial replacements for cement. However, as natural resources such as coal are being phased out, these disposals may not be a long-term solution. Limestone calcined clay cement (LC3), a novel concept that uses metakaolin, has been investigated as a partial replacement for cement by many scholars [13,14,15]. Their studies have shown that the mechanical performance and material properties of concrete containing LC3 as partial binders are at the same level as those of OPC concrete [16,17]. In principle, approximately one ton of CO_2_ is generated for every one ton of Portland cement produced, whereas the CO_2_ emission is reduced to approximately 0.3 tons for cement with LC3. In other words, around 30–40% of carbon dioxide emissions can be reduced in total when Portland cement is partially (25–50%) replaced by LC3.

**Figure 1:**
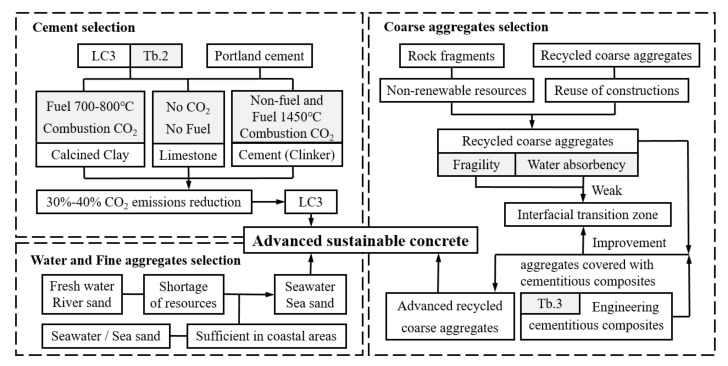
Composition of the advanced sustainable concrete.

Hence, in this study, an advanced alternative concrete containing LC3, seawater, sea sand, and recycled aggregates, was examined as a potentially sustainable solution to address the environmental issues, as shown in Figure 1. However, like normal concrete, the internal “glue” of the proposed advanced sustainable concrete (ASC) is also formed by a layer of calcium hydroxide, and a weak connection in the interfacial transition zone is likely to be present when under tension. Therefore, proper reinforcements are necessary for the ASC to increase tensile resistance. When steel, the most commonly used reinforcing material for concrete, is used in a chlorine-rich zone, it suffers from corrosion, thereby causing potential risks to the safety and durability of the structure. Fiber-reinforced polymer (FRP), on the other hand, is widely perceived as an alternative to steel reinforcement under an aggressive environment. Compared to steel, FRP composites are not only immune to corrosion but also offer extraordinary tensile resistance [18,19]. It is therefore finding use as fiber-reinforced concrete structures [20,21,22,23], strengthening jacket systems [24,25,26,27], or reinforcing bars [28,29,30,31,32,33,34,35,36,37,38,39] in infrastructure. Special attention should be given to FRP bars reinforced concrete, where the majority of the tensile load is expected to be carried by FRP bars. The implementation of FRP bar reinforcements not only reduces the crack and deflection, subsequently improving stiffness and strength of the structure, but also acts as the determination code for failure modes of the reinforced structure. The interchange of the major failure mechanisms, namely flexural and shear mode, can be determined by the design of the reinforcing bars. In the experimental investigations of CFRP reinforced slender beam, Karayannis et al. [33] reported that the cracking patterns shifted from flexural failure to shear failure when the reinforcing ratio of FRP bars increased. Maranan et al. and Tekle et al. compared the bonding performance of glass-fiber-reinforced polymer (GFRP) bars to that of steel bars in geopolymer concrete and found that the bond behavior of FRP bars was almost identical to the steel ones [35,37]. Deifalla et al. performed an experimental study on the torsion resistance of GFRP reinforced concrete beam and found that the use of GFRP reinforcements showed a significant improvement in terms of strength and toughness [32]. Abushanab et al. investigated the flexural behavior of basalt FRP bars strengthened concrete beam and found the reinforcement ratio was a major contributor to the strength of the beam [28]. Ahmed et al. conducted a comparison study on flexural resistance of two types of FRP (carbon-fiber-reinforced polymer (CFRP) and GFRP) strengthened geopolymer beams and found that the performance of the beams with CFRP bars showed better results in terms of ultimate load capacity and mid-span deflection compared to the GFRP bars [30]. Similar conclusions were made by [38], where CFRP bars were shown to have excellent performance for coral concrete beams. Bakar et al. reviewed the failure mechanisms and common analytical models for evaluating the flexural strength of concrete beams reinforced by CFRP bars [31]. Protchenko et al. examined the theoretical models and experimental results of FRP reinforced concrete beams and proposed a new model for predicting the ultimate bending capacity of reinforced beams [36]. Since recent achievement in the development of seawater and sea sand (SWSS) concrete, many researchers have made efforts to study FRP bars reinforced SWSS concrete structures. Zhang et al. performed a series of pull-out tests on the bonding behavior between FRP bars and SWSS concrete. In addition to the feasibility of application of FRP bars, they also found that the treatment of the interface between FRP bars and SWSS concrete was able to provide better adhesive performance between those two materials [39]. Liao et al. performed various types of pull-out tests to study the strength of the bond between GFRP bars and high-strength fiber-reinforced SWSS concrete structures. The results showed that the bonding properties of FRP bars to polyethylene (PE) fiber-reinforced SWSS concrete was promising compared to no fiber treatment [34]. An excellent review on the durability of FRP bars reinforced SWSS concrete was given by [29]. The authors found SWSS concrete structures reinforced with CFRP bars showed the best performance in terms of durability. According to the aforementioned findings, CFRP bars seem to be the optimal selection for reinforcing SWSS concrete structures. In addition, treatment of concrete, such as by PE fiber reinforcements, could significantly improve the bonding performance between FRP bars and concrete. To this end, in the current study, PE-fiber-reinforced engineering cementitious composites (ECC) were employed not only to enhance the bonding properties but also as a rigid shell to prevent the internally recycled aggregate from excess water absorbency and fragility.

Most of the aforementioned studies from the literature dealt with the bond of FRP–geopolymer concrete, FRP–SWSS concrete with normal cement, or FRP–fiber-reinforced concrete. To the best knowledge of the authors, there has been no previous work on the bond between FRP and the innovative ASC. The failure mechanisms, the relationship between the bonding stress and slip, and other parameters such as effectively confined embedment to diameter ratio remain to be elucidated. To this end, this study aimed to revisit the bonding performance of FRP bars in ASC, reveal the failure mechanisms and investigate the corresponding parameters, and describe the bond–slip relationship. To achieve this, pull-out tests were carried out to investigate the bond behavior of CFRP–recycled concrete, CFRP–SWSS concrete, and CFRP–ASC for comparison. Multiple bonding prediction models were evaluated, and a modified piecewise model was developed to describe details of the bond–slip relationship.

## 2. Materials and Methods

### 2.1. Material

The fresh water used here was obtained directly from tap water, and the seawater was acquired from the sea near Neilingding Island, Guangdong, China. The main ions of the fresh water and seawater are shown in Table 1. As can be seen, the content of chloride ions in the seawater was almost 200 times that of fresh water. The sea sand used was also obtained from Neilingding Island. The chloride ion content of the sea sand was 0.159%, the shell content was 0.4%, and the fineness modulus of the sea sand and river sand were 2.3 and 2.2, respectively. The Portland cement chosen in this study had the classification 42.5R, and LC3 was mainly composed of two materials: limestone and calcined clay (calcined kaolin with impure content, also known as metakaolin). Table 2 shows the chemical composition of LC3.

**Figure 2:**
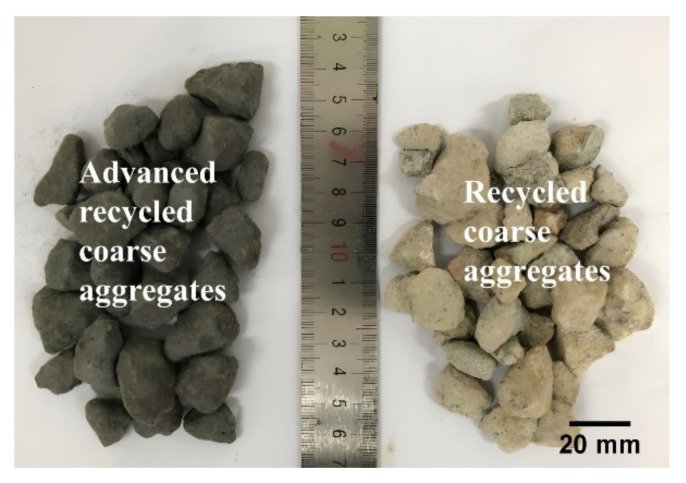
Coating appearance of RCA.

The recycled coarse aggregates were obtained from crushing construction waste. The particle size ranged from 5 to 20 mm, the corresponding water absorption capacity was 6.02%, the average apparent density was approximately 2610 kg/m^3^, and the average crushing index was measured as 19.52%. As shown in Figure 2, to prevent the recycled aggregate from excess water absorbency and fragility, PE-fiber-reinforced ECC was employed as a cover (wrapping layer) to the coarse aggregates. The mixing proportion of ECC is presented in Table 3. The weight ratio of ECC to recycled aggregates was set as 0.15, that is, 0.15 kg of ECC was applied for every one kilogram of aggregates.

The mix design for four types of concrete used in this study are listed in Table 4, where R represents advanced RCA with fresh water, Portland cement, and river sand; S stands for SWSS concrete with seawater and sea sand; and A(35) and A(50) represent ASC with 35 and 50% LC3 replacement, respectively. A compressive test was carried out using concrete cubes with specimens of 100 mm × 100 mm × 100 mm, a cylinder with diameter of 150 mm and height of 300 mm, and compressive strength of fcu,28d and fcy,28d. The diameters of the CFRP bars used in this study were all 12.7 mm. The material properties are shown in Table 5.

### 2.2. Specimen Preparation

A total of 36 specimens were prepared for 12 different conditions. Three specimens were tested for each condition to determine repeatability. The testing variables included the four types of concrete mentioned in the previous section and three different confined embedment thicknesses with the C/D ratios of 2.5, 3.5, and 4.5. The corresponding sample ID is listed in Table 6.

**Figure 3:**
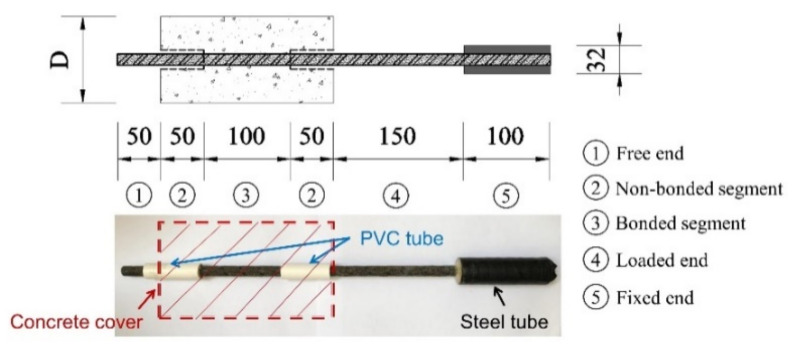
Schematic diagram of the specimen preparation.

A schematic diagram of the specimen preparation is shown in Figure 3. As can be seen, the height of the cylindrical concrete was 200 mm. The diameters of the concrete sections were 76.6, 101.6, and 126.6 mm, corresponding to the C/D ratios of 2.5, 3.5, and 4.5, respectively. The CFRP bar was aligned at the center of the concrete column, the bar length was 500 mm, and the embedded length was 100 mm. The effective embedment length was separated by two pieces of polyvinyl chloride (PVC) pipes of 50 mm length at both ends. To prevent the CFRP bars from being crushed by the grips of the testing machine, a steel pipe of 100 mm length was employed at the loading end, and expanded cement was injected into the gap between the steel and CFRP.

### 2.3. Test Setup and Instrumentation

**Figure 4:**
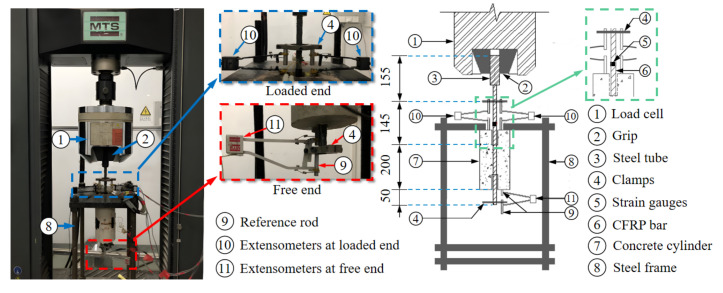
Pull-out test setup: photo and sketch.

The tests were conducted by utilizing a universal testing machine with a maximum tensile capacity of 300 kN. A steel frame was set up on the machine to hold the pull-out specimens. As shown in Figure 4, the lower part was a jig that held the specimen, while the upper part of the CFRP was clamped by the grip of the machine. A hole was drilled at the top of the jig to let the reinforcement bar protrude through the steel frame. Details of the setup at the loaded end and free end are shown in the schematic drawings. Displacement control was employed with a loading rate of 0.6 mm/min. The load was applied on the steel tube over the top part of the reinforcing bar, and the movement of the specimen was restrained by the bottom steel fixture. The top surface (contact surface at the loaded end) of the concrete cylinder was attached to the fixed steel plate before the test. The slips at both ends were measured using extensometers. The slip value at the free end was obtained as the measurement of the extensometer, while the slip at the loaded end was calculated as the difference between the extensometer and the predicted deformation of the CFRP. To eliminate the error of the slip at the loaded end, the deformation over the top section of the CFRP bar was considered. The predicted value was calculated as the product of the strain gauge reading and the effective length of the CFRP bar (Equation (2)). Data including force and vertical displacements were synchronously recorded by the data acquisition system.

## 3. Results and Discussions

### 3.1. Test Results

The bond strength in this study was defined as the shear load per unit surface area of the CFRP bar, which can be calculated as follows:

(1) $$\tau_{u}=\frac{F_{u}}{\pi dl},$$

where $\tau_{u}$ = bond strength in megapascals, $F_{u}$ = the maximum applied pull-out load in Newtons, d = the nominal diameter of the CFRP bar in millimeters, and l = the effective embedded length in millimeters.

The extensometers at the loaded end measured the variation in distance between a fixed perimeter of the bar and the concrete surface. Therefore, both the slip and the elongation of the bar were recorded. It was assumed that the elongation of the bar was distributed equally along the length of the CFRP. The length from the center of the clamp to the end of the bonding was measured as 0.145 m; hence, the loaded end slip needed to be adjusted by deducting the elongation of the bar outside the bonded region. The loaded end slip was therefore calculated as follows

(2) $$S_{r}=S_{m}-0.145\varepsilon ,$$

where $S_{r}$ = adjusted slip at the loaded end in millimeters, $S_{m}$ = slip measurement at the loaded end in millimeters, and ε = the measured axial strain of the CFRP bar. Details of the test results are summarized in Table 6.

### 3.2. Failure Modes

**Figure 5:**
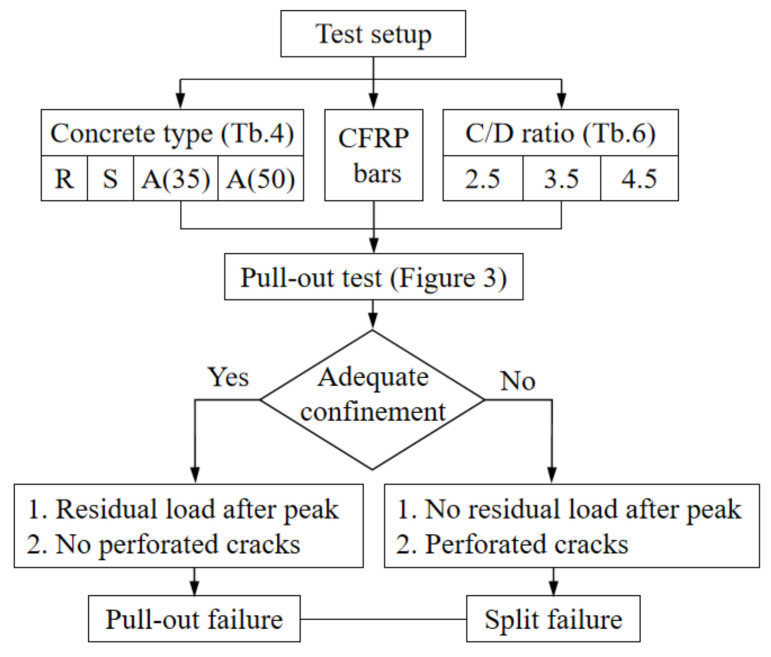
Experimental flowchart of failure mechanisms.

**Figure 6:**
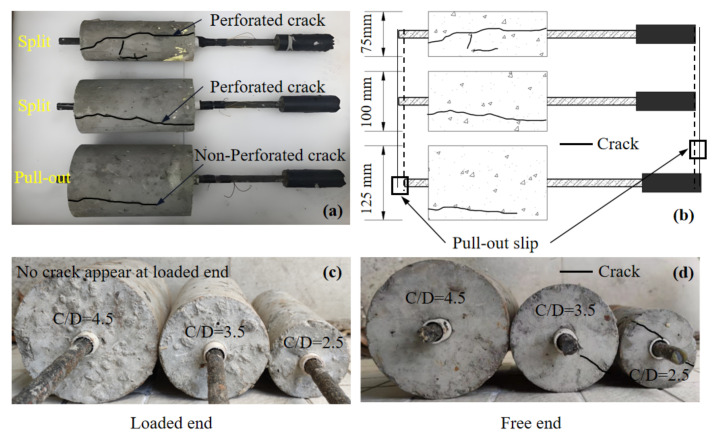
Failure modes of the specimens for different C/D ratios: (**a**,**b**) Top view; (**c**) Loaded end view; (**d**) Free end view.

A total of 36 pull-out tests were performed for four types of concrete and three confinement ratios. Two failure mechanisms, namely splitting and pull-out failure, were observed as a result of the confinement effect. The testing program was set according to the flowchart in Figure 5. The reflective failure modes are illustrated in Figure 6. As presented in Table 6, splitting failures only occurred for specimens with C/D ratios of 2.5 and 3.5 regardless of the type of concrete used. The main reason for this was that the confinement of the concrete was insufficient to provide enough restraint stress; hence, the splitting failure occurred along the longitudinal direction. At the early loading stage, no damage was observed. As the loading process continued, longitudinal perforated cracks were suddenly seen when the peak load was reached. However, due to the existence of PE fibers on the coating of the RCA, the specimens remained in one piece. This was in contrast to traditional concrete, where the concrete typically broke into several pieces.

As shown in Figure 6, pull-out failure only occurred on specimens with the C/D ratio of 4.5 regardless of the type of concrete. Instead of splitting failure, only a certain number of minor cracks were developed when peak load was reached. Only nonperforated cracks were found among the specimens. As the confinement was sufficient for the CFRP bar, pull-out failure occurred. As demonstrated in Figure 6c,d, the perforated cracks were only seen on the surface at the free end; the stress concentration at the free end was more evident than at the loaded end.

### 3.3. Bond Performance

**Figure 7:**
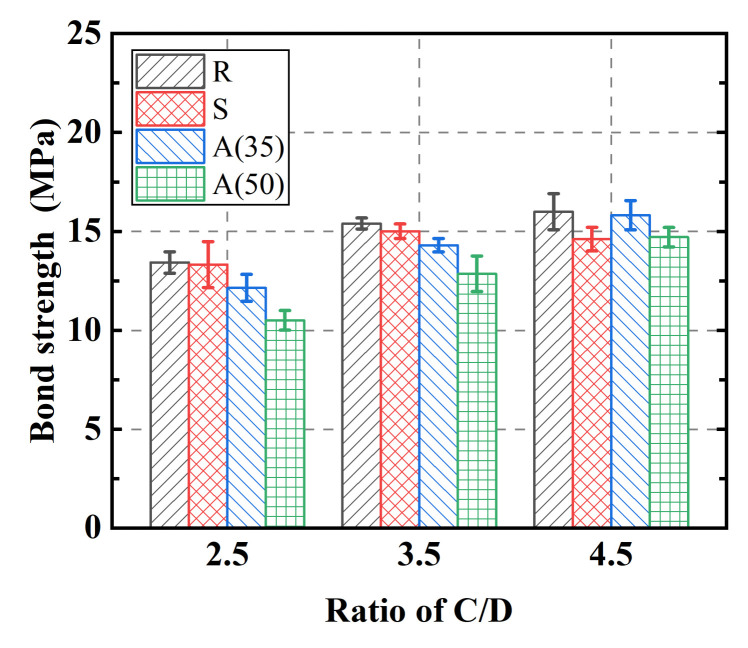
Comparison of average bond strength.

Figure 7 shows the average bond strength for the tested specimens. As can be seen, the restraining capacity showed an ascending trend with the increase in C/D ratio. The bond strength increased with the increase in confinement effect. When the ratio of C/D reached its extreme, somewhere between 3.5 and 4.5, the value of the bond strength reached its limit value, and no further increase was found in bond strength with increase in C/D ratio. Taking group R samples as an example, an increase of 14.6% was witnessed when the C/D ratio increased from 2.5 to 3.5, while these values increased only up to 3.2% when the C/D ratio increased from 3.5 to 4.5. As also confirmed from the failure mode, the critical value of the C/D ratio was between 3.5 and 4.5. The variation tendencies for the specimens in groups A(35) and A(50) were almost identical to other groups. However, a decrease in bond strength was seen for the SWSS concrete group. Regarding the type of concrete, the average values of the maximum bond strength increased approximately 14% when the C/D ratio increased from 2.5 to 3.5 regardless of the concrete type. The values for groups R and S (without the replacement of Portland cement) remained at the same level when the C/D increased from 3.5 to 4.5, while the value for groups A(35) and A(50) increased approximately 13% under the same circumstances. The critical value of the C/D ratio that distinguishes the split and pull-out failure modes varied with the change in cement material. The differences in variation of the maximum bond strength indicated that the critical values for groups R and S were close to 3.5, while the values for group A was close to 4.5. In addition, comparing groups R and S, the bond strength showed a descending pattern when seawater and sea sand were introduced. The values of bond strength decreased 2.8%, 2.6%, and 8.7% for C/D ratios of 2.5, 3.5, and 4.5, respectively.

**Figure 8:**
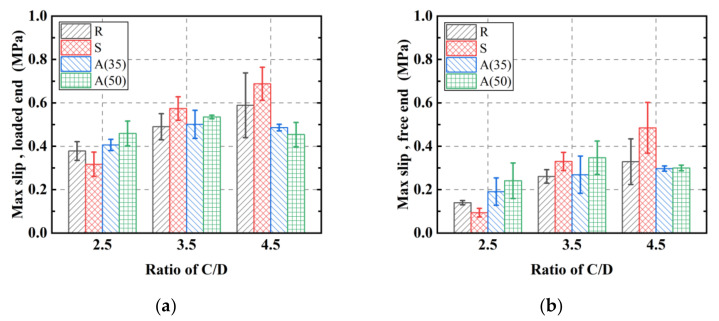
(**a**) Maximum slip at loaded end, (**b**) maximum slip at free end.

The average values of maximum slip at the loaded and free ends are illustrated in Figure 8. An identical growth trend was seen for the loaded and free ends. The maximum slip increased monotonically with the increase in C/D ratio. However, a slight decrease was captured for group A(50), with the value of slip decreasing when the ratio of C/D increased from 3.5 to 4.5. Regardless of the change in failure mode, the maximum slip kept increasing even when the maximum bond strength was reached.

### 3.4. Bond–Slip Curvatures

**Figure 9:**
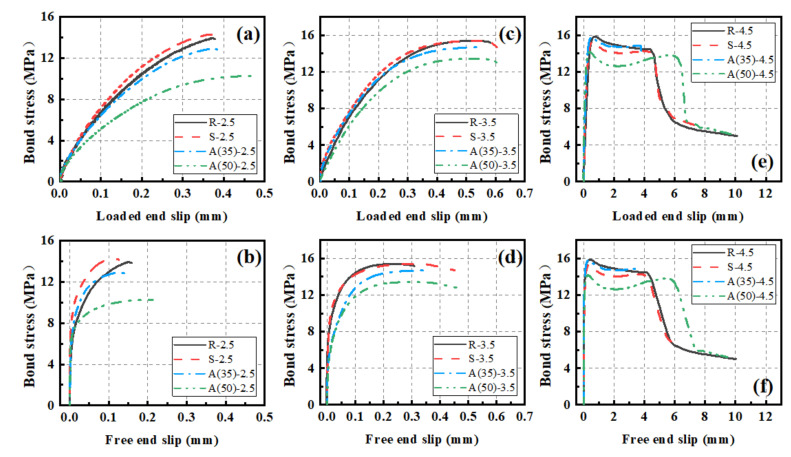
Bond–slip curves: (**a**) loaded end at ratio of 2.5, (**b**) free end at ratio of 2.5, (**c**) loaded end at ratio of 3.5, (**d**) free end at ratio of 3.5, (**e**) loaded end at ratio of 4.5, and (**f**) free end at ratio of 4.5.

The representative curvatures of the bond–slip relationship are demonstrated in Figure 9. As can be seen, the bond–slip curves of the loaded end exhibited a similar trend to that of the free end regardless of the type of concrete. A larger slope was seen in the early loading stage for the free end group. The difference mainly resulted from the lengthening of the reinforcing bar between the two points of the slip measurements. The slip at the loaded end lagged that of the free end, indicating the initiation of nonlinear bond stress across the embedment length of the CFRP.

**Figure 10:**
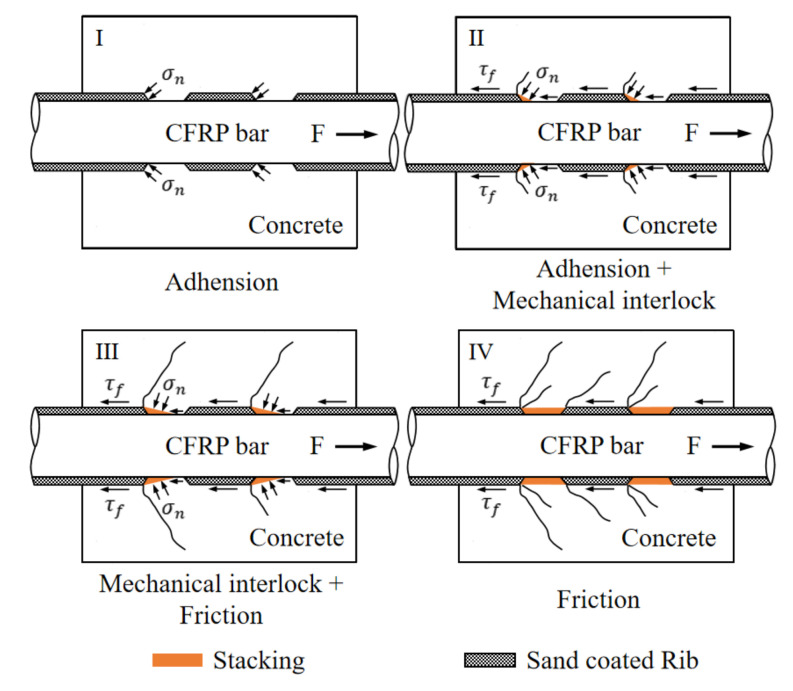
Schematic diagram of failure mechanisms at different stages.

As illustrated in Figure 9, the typical bond–slip curves could be divided into four stages: hardening stage, softening stage, descending stage, and residual stage. The hardening stage was seen for all the specimens regardless of the C/D ratio. In the early hardening stage, the load showed a linear increase up to approximately 8 MPa. The bond was resisted mainly by the adhesion between the two materials of CFRP and concrete. Thereafter, a nonlinear increase in stress was seen up to the peak value. During this period, the adhesion became weakened, and the bond was resisted mainly by the mechanical interlock due to existence of the ribs of the reinforcements. For the C/D ratio of 2.5, a major crack developed along the edge of the rib and propagated through the concrete section, resulting in splitting failure. The second stage saw the stress remain at a certain level while the slip still increased. As the loading process continued, a small amount of concrete was stacked by the rib. The resistance provided by the mechanical interlock was weakened, thereby resulting in softening of the stiffness. For the C/D ratio of 3.5, besides the minor cracks initiated at the edges of the interlock, a major crack developed along the edge of the stacking location and perforated through the concrete section, resulting in splitting failure. When the CFRP bar was restrained sufficiently by the depth of the concrete, the third stage showed a significant drop in stress. In this stage, the resistance provided by the interlock gradually faded away along with the increase in stacking. The bond was resisted mainly by friction. The fourth and last stage saw the process of complete pull-out. The bond was only resisted by the remaining friction. A detailed schematic diagram is shown in Figure 10 to explain the development of the bond resistance for the four-stage loading process.

## 4. Analytical Models for Bond–Slip Relationship

### 4.1. Prediction of the Ultimate Bond Strength

In light of the above background, a complete bond–slip relationship of FRP–ASC can be expressed in four stages. In particular, the boundary of the stage between the first two stages is defined as having the ultimate bond strength. Hence, the accuracy of the bond–slip constitutive model is profoundly influenced by the prediction of the ultimate state.

In this study, three representative prediction models were examined to determine the best fitting model for the prediction of the maximum bond strength. Many previous studies have reported that the bond strength of FRP or steel bar is a function of the compressive strength of the confined concrete [40,41,42]. Two surface parameters of $\alpha^{\prime }$ and β were introduced for various surface conditions of the reinforcing bars. The model is expressed as follows:

(3) $$\tau_{\max}=\alpha^{\prime }{\left(f_{c}\right)}^{\beta },$$

where $\tau_{\max}$ = the predicted maximum bond strength, $\alpha^{\prime }$ and β are the surface parameters, and $f_{c}\;$= the compressive strength of cylindrical concrete. More recently, with the development of FRP and other types of reinforcing elements, Nepomuceno et al. summarized a new database for the reference values of the surface parameters [43]. The values of these two surface parameters were recommended as 3.33 and 0.41, respectively, for sand-covered CFRP surface.

The other two prediction models suggest that the bond strength is not only set as a function of compressive strength but also correlated with other parameters, such as the C/D ratio and the ratio of the diameter to the effective embedded length [44,45]. The prediction models recommended by ACI 440.1R-06 (2006) and Aslani and Nejadi (2012) are expressed in Equations (4) and (5), respectively [44,45].

(4) $$\tau_{\max}=\;0.083\sqrt{f_{c}}\left(4.0+0.3\frac{c}{d_{b}}+100\frac{d_{b}}{l_{b}}\right),$$

(5) $$\tau_{\max}=\left(0.672{\left(\frac{c}{d_{b}}\right)}^{0.6}+4.8\frac{d_{b}}{l_{b}}\right)f_{c}{}^{0.55},$$

where $\tau_{\max}$ = the predicted maximum bond strength, $f_{c}$ = the compressive strength of cylindrical concrete, $\frac{c}{d_{b}}$ = the ratio of the cover depth to the diameter of the bar, and $\frac{d_{b}}{l_{b}}$ = the ratio of the diameter to the effective embedded length.

**Figure 11:**
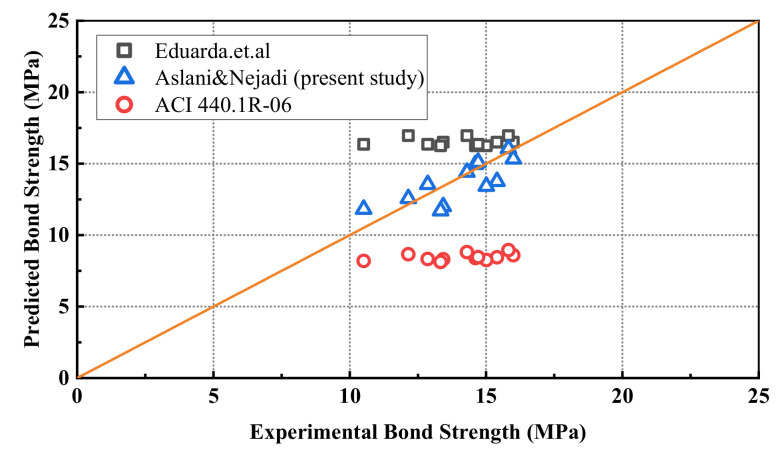
Comparison of the predicted maximum bond strength.

To examine the feasibility of the proposed models, experimental data obtained in this study were used to derive the bond strength using each of the selected bond strength equations. For ease of calculation, the specimens were grouped, and an average value of experimental bond strength and compressive strength was used for identical specimens. The values of the predicted bond strength from the corresponding model are given in Figure 11. As can be seen, the model developed by Aslani and Nejadi gave the best fitting results for the experimental data in this study. This prediction method is utilized to present the reference value of the maximum bond strength for the constitutive bond–slip model in the next section.

### 4.2. Bond–Slip Constitutive Model

The two most popular bond–slip models in the literature are the Eligehausen, Popov, and Bertero (BPE) model [46] and the CMR model [47]. As mentioned above, a constitutive bond–slip model of FRP–ASC should be able to describe all four stages of the loading process. As the CMR law is only able to describe the ascending branch (single stage), a modified BPE (mBPE) model is presented in this study. To describe all four stages of the bond behavior of CFRP–ASC, a four-segment expression is proposed corresponding to the hardening stage, softening stage, descending stage, and residual stage:

(6) $$\tau =\{\begin{array}{ll} (S/S_{1}{)}^{\alpha }\tau_{1}, & S\leq S_{1}\\ \tau_{1}, & S_{1}<S\leq S_{2}\\ \tau_{1}-(S_{2}/S)(\tau_{1}-\tau_{3})/(S_{2}-S_{3}), & S_{2}<S\leq S_{3}\\ \tau_{3}, & S>S_{3} \end{array},$$

where τ = the present bond strength, S = the present slip, $S_{1}$ = maximum slip in stage one, α = surface type parameter, $\tau_{1}$ = maximum bond strength, $S_{2}$ = maximum slip in stage two, $\tau_{3}$ = residual bond strength, and $S_{3}$ = maximum slip in stage three.

**Figure 12:**
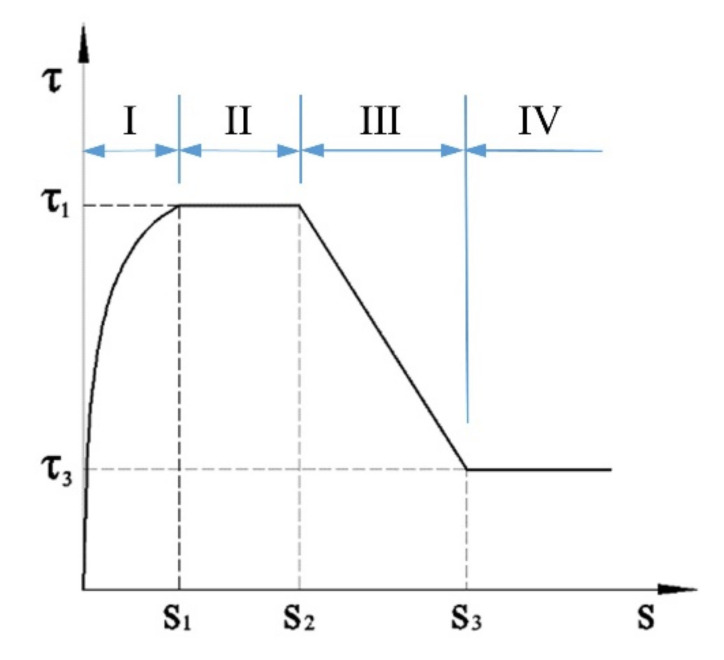
Schematic diagram of the constitutive model (I: Hardening stage; II: Platform; III: Softening stage; IV: Residual stage).

For the hardening stage, the value of $\tau_{1}$ can be calculated from Equation (5). In a more recent study, the values of the surface parameter α were recommended as 0.493 and 0.134, respectively, for the loaded end and free end of sand-coated FRP bars. The consequent maximum slip in the first stage was also given as 0.495 and 0.281, respectively, for the loaded end and free end [48]. For the softening stage, a differential value of 4 mm between the $S_{2}$ and $S_{1}$ was recommended for the platform sectional value of sand-coated FRP bars in the pull-out tests [49]. Hence, the values of $S_{2}$ were calculated as 4.495 and 4.281 for the two ends. For the third and last stage, the residual strength of the sand-coated FRP bars was advised as 40% of the maximum bond strength in [50], while the slip at the end of the third stage can be assumed to be equal to the value of rib spacing. Therefore, a value of 6 mm was set for $S_{3}$ as standard. Details of the corresponding input parameters are listed in Table 7. A schematic diagram of the constitutive bond–slip model is given in Figure 12.

**Figure 13:**
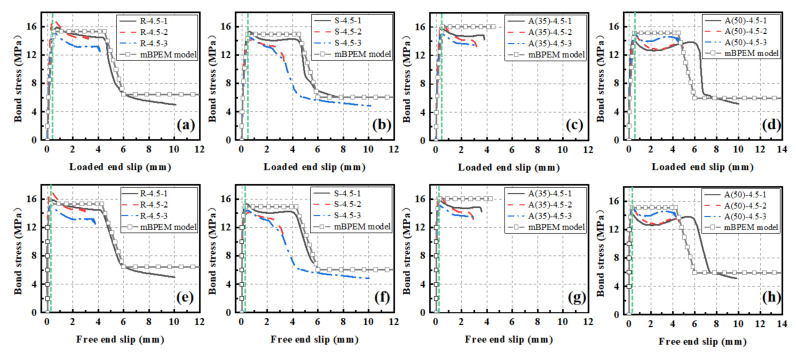
Comparison of bond–slip relationships: (**a**) loaded end of group R-4.5, (**b**) loaded end of group S-4.5, (**c**) loaded end of group A(35)-4.5, (**d**) loaded end of group A(50)-4.5, (**e**) free end of group R-4.5, (**f**) free end of group S-4.5, (**g**) free end of group A(35)-4.5, and (**h**) free end of group A(50)-4.5.

Figure 13 shows verification for the proposed bond–slip model. The predicted results were compared with the experimental ones, and a reasonably good agreement was achieved between them. As can be seen, in the first (hardening) stage, the free end bond–slip curve showed lower stiffness than the loaded end. This behavior was well predicted for both ends. In the platform (softening) stage, the amount of total slip (the difference between $S_{2}$ and $S_{1}$) that occurred was also well predicted by the proposed model. However, a slight decrease was seen in the experimental data. This reduction in bond stiffness was not expected and considered in the analytical model. The analytical results were in agreement with the experimental ones in the third and last stage. Approximately 40% of the maximum bond strength was expected as the residual bond. One thing to note is that the third and fourth stages were missing for group A(35) with 35% LC3 replacement. Here, the confinement strength was insufficient to sustain the bar from complete pull-out failure.

## 5. Conclusions

This study reports on the results of experimental and analytical investigations aimed at exploring the bond behavior of FRP and innovative sustainable concrete. Parameters such as C/D ratio and type of concrete were examined. The results in terms of bond strength, slip, and bond stiffness were evaluated and compared. A modified BPE model was developed to describe the four-stage bond–slip relationship. Based on the results and discussions presented, the following conclusions can be drawn:

1. Two major failure modes were seen for different levels of confinement. Splitting failure typically occurred when the confinement was insufficient. The critical value of C/D ratio was determined as being between 3.5 and 4.5. This value was close to 3.5 for groups R and S and 4.5 for groups A(35) and A(50). Further work is required to determine this critical value.
2. The bond performance in terms of maximum slip increased monotonically with the increase in confinement, while the bond strength increased until the critical ratio of C/D was reached. For the group with 50% LC3 replacement, lower bond strength was seen when the confinement was insufficient. Comparing groups R and S, the bond strength showed a descending pattern when seawater and sea sand were introduced.
3. When the confinement was adequate, the bond–slip relationship between sand-coated CFRP bar and ASC was represented by a four-stage process. The variation tendencies presented the combined bonding mechanisms of the internal adhesion, mechanical interlock, and friction.
4. Multiple ultimate bond strength models were evaluated. The bond strength of sand-coated CFRP reinforced ASC were dependent on the compressive strength, confinement effect, and effective embedded length. A modified BPE model was developed to describe the constitutive relationship between CFRP bar and ASC. The predictions were found to corroborate with the experimental results. A similar bond behavior was found regardless of the type of concrete. The innovative concrete with 35 or 50% LC3 replacement, seawater, sea sand, and treated recycled aggregates exhibited almost identical bond behavior to other types of concrete.

The parameters that affect the bond behavior are not limited to the ratio of confinement to bar diameter and concrete type. Other parameters, such as the type of reinforcement, surface conditions, and embedded length, can also make an impact on the bond performance of CFRP reinforced ASC. Indeed, in real-life application, the bond behavior of FRP strengthened ASC typically involves a complicated combination of the aforementioned factors. Hence, other potential factors need to be examined to further expand on the findings of this study. Future works are expected to explore correlation analysis of the relevant parameters.

## Figures and Tables

**Table 1:** Main ions in the fresh water and seawater.

Type	Unit	Fresh Water	Seawater
Na^+^	mg/L	2.69	4.24 × 10^3^
K^+^	mg/L	11.20	380
Ca^2+^	mg/L	6.65	390
Mg^2+^	mg/L	1.76	1.25 × 10^3^
F^−^	mg/L	0.17	<0.10
Cl^−^	mg/L	10.90	1.97 × 10^3^
SO_4_^2−^	mg/L	7.19	5.24 × 10^3^
CO_3_^2−^	mg/L	<0.10	11.78

**Table 2:** Chemical composition of LC3.

Oxide	Cement (%)	Metakaolin (%)	Limestone (%)
SiO_2_	20.271	53.732	0.309
Al_2_O_3_	5.184	39.405	0.130
K_2_O	0.809	4.229	0.040
Fe_2_O_3_	4.087	2.056	-
MgO	0.086	0.307	0.769
TiO_2_	0.349	0.18	-
SO_3_	2.684	0.087	-
CaO	63.913	0.102	98.715
Rb_2_O	-	0.037	-
SrO	0.030	-	0.037
MnO	0.098	-	-
ZnO	0.079	-	-
P_2_O_5_	0.044	0.037	-
Na_2_O	0.086	-	-

**Table 3:** Mixing proportion of ECC.

C/R Ratio	RCA	Water	Cement	Fly Ash	PE Fiber	Superplasticizer	Alkali-Free Accelerator
0.15	1	0.058	0.113	0.038	1.416 × 10^−^^3^	2.123 × 10^−3^	3 × 10^−^^3^

**Table 4:** Concrete mixture proportions (kg/m^3^).

Concrete	ARCA	Water	Sand	Cement	Metakaolin	Limestone	f_cu,28d_ (MPa)	f_cy,28d_ (MPa)
R	1070	274	576	538	-	-	36.20	32.33
S	1070	274	576	538	-	-	31.92	30.89
A(35)	1070	274	576	360	119	59	38.48	35.14
A(50)	1070	274	576	296	161	81	36.56	31.43

**Table 5:** Properties of CFRP bar.

Surface	Diameter (μm)	Ultimate Tensile Strength (MPa)	Transverse Shear Strength (MPa)	Ultimate Strain (%)	Elastic Modulus (GPa)
HWSC ^1^	12.7	2345	231	2.0	135

^1^ HWSC: helically wrapped and sand coated.

**Table 6:** Summary of the test results.

Specimen Symbol	Max Bond Load (kN)	Max Bond Stress (MPa)	Max Slip, Loaded End (mm)	Max Slip, Free End (mm)	Failure Mode
R-2.5	53.94	13.52	0.423	0.130	Split
	51.31	12.86	0.337	0.139	Split
	55.54	13.92	0.374	0.150	Split
R-3.5	61.40	15.39	0.521	0.236	Split
	60.36	15.13	0.461	0.296	Split
	62.56	15.68	0.487	0.252	Split
R-4.5	63.20	15.84	0.761	0.447	Pull-out
	67.75	16.98	0.494	0.294	Pull-out
	60.56	15.18	0.511	0.247	Pull-out
S-2.5	48.00	12.03	0.257	0.100	Split
	56.85	14.25	0.367	0.111	Split
	54.70	13.71	0.327	0.072	Split
S-3.5	58.57	14.68	0.628	0.376	Split
	61.48	15.41	0.521	0.293	Split
	59.57	14.93	0.573	0.320	Split
S-4.5	61.00	15.29	0.619	0.389	Pull-out
	56.45	14.15	0.770	0.615	Pull-out
	57.41	14.39	0.676	0.452	Pull-out
A(35)-2.5	47.00	11.78	0.411	0.189	Split
	46.80	11.73	0.430	0.254	Split
	51.63	12.94	0.378	0.129	Split
A(35)-3.5	56.53	14.17	0.516	0.284	Split
	58.61	14.69	0.557	0.347	Split
	56.02	14.04	0.429	0.176	Split
A(35)-4.5	66.11	16.57	0.474	0.283	Pull-out
	63.04	15.80	0.503	0.305	Pull-out
	60.24	15.10	0.480	0.303	Pull-out
A(50)-2.5	40.66	10.19	0.509	0.334	Split
	40.89	10.25	0.472	0.213	Split
	44.21	11.08	0.397	0.177	Split
A(50)-3.5	53.18	13.33	0.539	0.303	Split
	53.58	13.43	0.526	0.303	Split
	47.16	11.82	0.541	0.436	Split
A(50)-4.5	59.25	14.85	0.495	0.308	Pull-out
	60.28	15.11	0.477	0.306	Pull-out
	56.53	14.17	0.391	0.285	Pull-out

**Table 7:** Input parameters for the mBPE Model.

**Model**	α	$S_{1}$	$\tau_{1}$	$S_{2}$	$S_{3}$	$\tau_{3}$
Loaded end	0.493	0.495	Equation (5)	4.495	6	0.4$\tau_{1}$
Free end	0.134	0.281	Equation (5)	4.281	6	0.4$\tau_{1}$

## Data Availability

Data is available on request.
